# Draft Genome Sequence of NDM-Encoding Klebsiella pneumoniae Isolated from Feral Swine

**DOI:** 10.1128/MRA.00808-21

**Published:** 2021-10-14

**Authors:** Huijia Liu, Yuting Zhai, Ting Liu, Peixin Fan, Raoul Boughton, Kwangcheol C. Jeong

**Affiliations:** a Emerging Pathogens Institute, University of Florida, Gainesville, Florida, USA; b Department of Animal Sciences, College of Agricultural and Life Sciences, University of Florida, Gainesville, Florida, USA; c The Mosaic Company, Lithia, Florida, USA; University of Maryland School of Medicine

## Abstract

New Delhi metallo-β-lactamase (NDM)-producing *Enterobacteriaceae* pose a great threat to public health globally. Most known NDM-producing *Enterobacteriaceae* are associated with human hospital or community infections. Here, we report the draft genome sequence of an NDM-1-encoding Klebsiella pneumoniae strain isolated from feral swine (Sus scrofa) captured in Florida, USA.

## ANNOUNCEMENT

New Delhi metallo-β-lactamase-1 (NDM-1) is a transferable molecular class B (zinc metallo-) β-lactamase which hydrolyzes beta-lactam antibiotics, including all penicillins, cephalosporins, and carbapenems (1). NDM-1 was first identified in K. pneumoniae and E. coli strains, from a Swedish patient with a hospitalization history in India in 2008 (2), and has been isolated from various hosts, including human, livestock, and companion animals (3, 4). As part of a large collaborative project on feral swine ecology, we collected 393 fecal samples from feral swine captured on Archbold’s Buck Island Ranch (27°09′N, 81°11′W). All activities associated with the trapping and sampling of feral swine occurred under Institutional Care and Use Committee-approved protocols (201408495, 201808495). Fecal samples (0.1 g) were incubated in tryptic soy broth containing meropenem (16 μg/ml) overnight at 37°C; then, resistant bacteria were selected on MacConkey agar containing meropenem (16 μg/ml). Meropenem-resistant isolates were subjected to PCR after boiling lysis to detect the *bla*_NDM_ gene using primer pairs (forward: 5′-GGTTTGGCGATCTGGTTTTC-3′; reverse: 5′-CGGAATGGCTCATCACGATC-3′) as described previously (5). One strain (KCJ2K2161) was identified as NDM positive and applied for whole-genome sequencing.

KCJ2K2161 was cultured overnight at 37°C in tryptic soy broth containing meropenem (16 μg/ml). Genomic DNA was extracted using the DNeasy blood and tissue kit (Qiagen, Valencia, CA), and the DNA concentration was measured using a Qubit 3 fluorometer (Invitrogen, Waltham, MA). To construct a DNA library, the DNA sample was diluted and quantified to 0.2 to 0.4 ng/μl. The DNA library was prepared using the Nextera XT sample preparation kit following the manufacturer’s instructions (Illumina, San Diego, CA). Genome sequencing was performed using the Illumina MiSeq platform with a 2 × 250-bp, 500-cycle cartridge (Illumina). The total number of reads and coverage of KCJ2K2161 were 1,581,846 and 74×, respectively. Sickle v1.33.2 (6) was used for adaptive trimming of the FASTQ raw sequencing data, with the quality and length thresholds set to 30 and 50 bp, respectively. Genome assembly was performed using SPAdes v3.12.0 (7). In the SPAdes genome assembler, the K-mer used was set as 21, 33, 55, 77, 99, 127, and the coverage cutoff was set as auto. Contigs that are less than 200 bp were removed. QUAST v5.0.2 (8) was used to evaluate the genome assembly quality. The assembled genome size of KCJ2K2161 was 5,340,476 bp, containing a total of 278 contigs, with a GC content of 57.09% and an *N*_50_ value of 107,917 bp.

KCJ2K2161 was identified as Klebsiella pneumoniae sequencing type 1967 using SpeciesFinder v2.0 and MLST v2.0 (9, 10). The genome sequence of K. pneumoniae KCJ2K2161 was annotated using the NCBI Prokaryotic Genome Annotation Pipeline v5.2. A total of 5,324 coding sequences, 84 tRNAs, 6 complete rRNAs, 11 noncoding RNAs, and 141 pseudogenes were identified. Using Resistance Gene Identifier v5.2.0 within the Comprehensive Antibiotic Resistance Database v3.1.3 (11), a total of 38 antibiotic-resistant genes were identified, including four beta-lactamase genes (*bla*_TEM-1_, *bla*_NDM-1_, *bla*_SHV-26_, and *bla*_AmpH_). A total of 14 virulence factors related to adherence, invasion, and iron uptake were detected using the PathoSystems Resource Integration Center v3.6.9 (12). Default parameters were used for all software unless otherwise specified.

### Data availability.

This whole-genome shotgun project has been deposited in DDBJ/ENA/GenBank under the accession number JAHWDE000000000. The version described in this paper is version JAHWDE010000000. The reads are available through the NCBI Sequence Read Archive under accession number SRR15327870.
